# Respiratory Syncytial Virus Protects Bystander Cells against Influenza A Virus Infection by Triggering Secretion of Type I and Type III Interferons

**DOI:** 10.1128/jvi.01341-22

**Published:** 2022-11-03

**Authors:** Maciej Czerkies, Marek Kochańczyk, Zbigniew Korwek, Wiktor Prus, Tomasz Lipniacki

**Affiliations:** a Institute of Fundamental Technological Research, Polish Academy of Sciences, Warsaw, Poland; Hudson Institute of Medical Research

**Keywords:** influenza A virus, innate immunity, interferon beta, interferon lambda, respiratory syncytial virus, single cell, viral interference

## Abstract

We observed the interference between two prevalent respiratory viruses, respiratory syncytial virus (RSV) and influenza A virus (IAV) (H1N1), and characterized its molecular underpinnings in alveolar epithelial cells (A549). We found that RSV induces higher levels of interferon beta (IFN-β) production than IAV and that IFN-β priming confers higher-level protection against infection with IAV than with RSV. Consequently, we focused on the sequential infection scheme of RSV and then IAV. Using A549 wild-type (WT), IFNAR1 knockout (KO), IFNLR1 KO, and IFNAR1-IFNLR1 double-KO cell lines, we found that both IFN-β and IFN-λ are necessary for maximum protection against subsequent infection. Immunostaining revealed that preinfection with RSV partitions the cell population into a subpopulation susceptible to subsequent infection with IAV and an IAV-proof subpopulation. Strikingly, the susceptible cells turned out to be those already compromised and efficiently expressing RSV, whereas the bystander, interferon-primed cells are resistant to IAV infection. Thus, virus-virus exclusion at the cell population level is not realized through direct competition for a shared ecological niche (single cell) but rather is achieved with the involvement of specific cytokines induced by the host’s innate immune response.

**IMPORTANCE** Influenza A virus (IAV) and respiratory syncytial virus (RSV) are common recurrent respiratory infectants that show a relatively high coincidence. We demonstrated that preinfection with RSV partitions the cell population into a subpopulation susceptible to subsequent infection with IAV and an IAV-proof subpopulation. The susceptible cells are those already compromised and efficiently expressing RSV, whereas the bystander cells are resistant to IAV infection. The cross-protective effect critically depends on IFN-β and IFN-λ signaling and thus ensues when the proportion of cells preinfected with RSV is relatively low yet sufficient to trigger a pervasive antiviral state in bystander cells. Our study suggests that mild, but not severe, respiratory infections may have a short-lasting protective role against more dangerous respiratory viruses, including severe acute respiratory syndrome coronavirus 2 (SARS-CoV-2).

## INTRODUCTION

Human respiratory tract infections are predominantly caused by viruses. Out of a plethora of respiratory viruses, recently expanded by severe acute respiratory syndrome coronavirus 2 (SARS-CoV-2) (1), the three most common recurrent infectants, originating from different viral families, are rhinovirus (RV) (human RV [HRV]), influenza A virus (IAV), and respiratory syncytial virus (RSV) (human orthopneumovirus) (2), with the latter being the most common pathogen of severe disease in young children and the elderly (3–5). In temperate areas of the Northern Hemisphere, the incidence of respiratory infections surges in the winter season and subsides during the summer. Detailed examination of temporal patterns of disease outbreaks reveals not only seasonal coincidence but also the apparent avoidance of some types of cocirculating respiratory viruses (6–9). It is unclear whether the epidemiological observations are population-level consequences of the interplay of multiple viral cooperation and competition mechanisms that operate at the single-host level (10). Intriguingly, the epidemiological avoidance patterns are also visible for taxonomically different groups of viruses, suggesting their potential reliance on mechanisms that are not related to specific antibody-mediated cross-protection. Although viral interference can be readily observed *in vitro* (11), its underlying mechanisms are necessarily inextricably intertwined with the response of the host organism and await elucidation from a systemic perspective.

Nonantigenic competitive interactions among respiratory viruses have been suggested based on clinical data analyses (12, 13) and were recapitulated within animal models (14, 15). Two very recent studies (16, 17) demonstrated that an innate immunity mechanism, specifically antiviral cytokine signaling, is implicated in the interference between RV and IAV: blocking the type I interferon (IFN) response restores IAV replication following RV infection. Whereas type I IFNs (IFN-α and IFN-β) were originally discovered specifically because of their activity against influenza virus (18), their roles in RSV infection and disease have been controversial (19–25). A recent animal model study (14) suggested that RSV-IAV interference is antigen-independent; however, the direct involvement of IFNs has not been demonstrated.

Type I IFNs, together with the more recently identified type III IFNs (IFN-λ1 to IFN-λ4) (26, 27), are produced and secreted by infected cells to trigger the transcription of an array of IFN-stimulated genes (ISGs) in both infected and bystander cells (28–30). Elevated ISGs restrict viral replication until virus-specific host defense mechanisms develop (31). IFN-α and IFN-β additionally attract specific immune system cells to the site of inflammation (22, 32). IFN-λ acts more locally, preferentially at epithelial barriers (33), and differentially affects tissue homeostasis: while IFN-λ confers longer-lasting protection than IFN-α (34), chronic IFN-λ exposure inhibits the proliferation of respiratory epithelial cells, impairing postinfection lung regeneration (35, 36). The significant relative potency of IFN-λ in the induction of an antiviral state in response to IAV has been demonstrated (34, 37–40). In the absence of IFN-β, IFN-λ has been proposed to be the primary factor preventing RSV infection (25).

The propagation of RSV strongly depends on the expression of its two nonstructural proteins, NS1 and NS2, which, among multiple modalities of interference with the innate immune response, block the synthesis of interferons and downregulate the expression of ISGs (by inhibition of STAT1/2 signaling) (41, 42). Of note, the strength of the immune response to RSV increases with the contents of the immunostimulatory defective viral genomes (DVGs), typically incomplete viral progeny that can stimulate the production of interferons (43, 44). DVGs are naturally generated during RSV replication and may limit the spread of RSV as well as other coinfecting viruses. *In vitro*, at low multiplicities of infection (MOIs), when the selective pressure on the virus is high, the incidence of DVGs is low (43). The kinetics of DVG accumulation may predict the clinical outcome of RSV infection in humans; the detection of DVGs early after infection has been associated with low viral loads and mild disease (45).

In this study, we sought to determine the molecular underpinnings of the interactions between RSV and IAV. We used a human alveolar epithelial cell line (A549) and characterized direct interviral relationships and interferon-mediated interference *in vitro* at the single-cell level. We found that RSV induces higher-level IFN-β production than IAV and that IFN-β is a more potent inducer of STAT1/2 signaling than IFN-λ1. Moreover, IFN-β-stimulated cells are more resistant to infection with IAV than to infection with RSV. In light of these findings, we focused on a sequential coinfection scheme in which cells are preinfected with RSV and then infected with IAV. We developed A549-derived cell lines with a knockout (KO) of the type I IFN receptor (IFNAR1 KO) or the type III IFN receptor subunit (IFNLR1 KO) and a double KO (dKO) (IFNAR1-IFNLR1 dKO). These cell lines enabled us to find that IFN-β is the main inducer of an antiviral state in an RSV-infected population of A549 cells and that both IFN-β and IFN-λ are simultaneously necessary for building maximum protection against subsequent infection with IAV. Immunostaining revealed that preinfection with RSV partitions the cell population into a subpopulation susceptible to subsequent infection with IAV and an IAV-proof subpopulation. Strikingly, the susceptible cells turned out to be those already compromised and efficiently expressing the priming virus. Overall, we conclude that in the sequential RSV-then-IAV infection scheme, virus-virus exclusion at the cell population level is not realized through direct competition for a shared ecological niche (single cell) but rather is achieved with the involvement of specific cytokines induced by the host’s innate immune response.

## RESULTS

### IFN-β and IFN-λ mediate STAT1/2 activation upon RSV and IAV infection.

**FIG 1:**
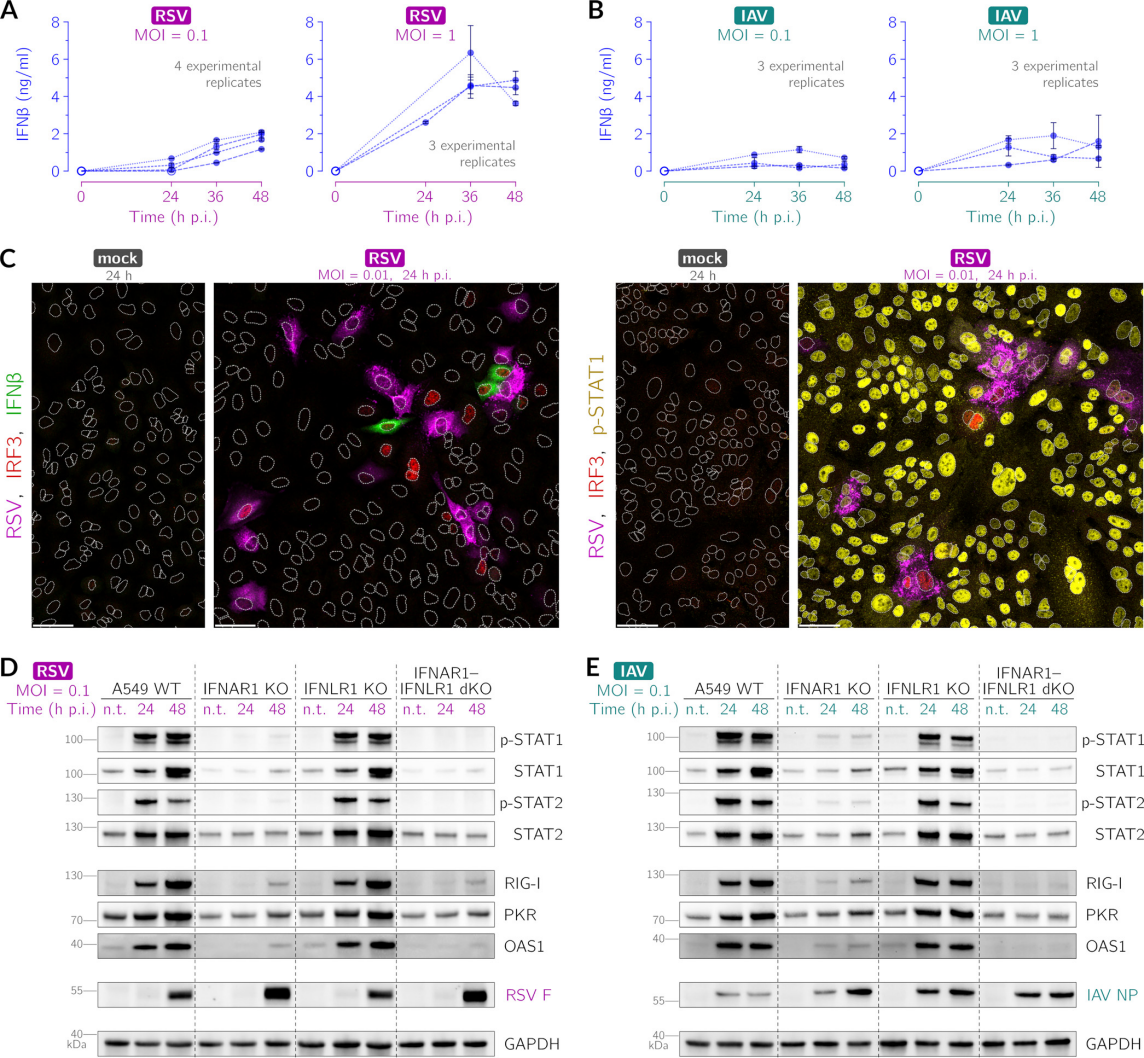
IFN-β and IFN-λ mediate STAT1 and STAT2 activation upon RSV and IAV infection in A549 cells. (A and B) ELISA for IFN-β following RSV (A) and IAV (B) infection at MOIs of 0.1 and 1. Open circles mark data points with cytokine concentrations below the detection range. Error bars are standard deviations (SD) from technical replicates. (C) IFN-β (green) (left) and activation of STAT1 (yellow) (right) and IRF3 (red) 24 h after infection of A549 WT cells with RSV (magenta, polyclonal anti-RSV antibody) at an MOI of 0.01. White dotted lines are nuclear outlines determined based on DAPI counterstaining (channel not shown). Bars, 50 μm. (D and E) Activation of STAT1, STAT2, and interferon-stimulated genes (RIG-I, PKR, and OAS1) upon RSV (D) and IAV infection (E) at an MOI of 0.1 at 24 and 48 h postinfection (p.i.) in A549 WT, IFNAR1 KO, IFNLR1 KO, and IFNAR1-IFNLR1 dKO cells. n.t. indicates nontreated cells. Activated STATs p-STAT1 and p-STAT2 are STAT1 and STAT2 phosphorylated at Tyr701 and Tyr690, respectively. RSV F, RSV fusion glycoprotein; IAV NP, IAV nucleoprotein.

We first characterized the induction of an antiviral state by interferon signaling. We found that RSV is a more potent inducer of IFN-β than IAV at comparable MOIs during 48 h after infection (cf. Fig. 1A and B). As shown in Fig. 1C, upon infection with RSV at a low MOI, only a tiny fraction of cells (1 to 5%) produces IFN-β, which, however, is sufficient to activate the STAT pathway in the remaining cells. Cells expressing IFN-β typically do not express RSV proteins, which may result from the early stage of infection, the suppression of RSV protein expression by the innate immune response in infected cells, or infection by DVGs. At the same time, in cells that express RSV proteins, nonstructural proteins may inhibit the synthesis of IFN-β at various levels (46–50). The detection of viral RNA within an RSV-infected cell is manifested by the activation of IFN regulatory factor 3 (IRF3) (which is phosphorylated and then translocates to the nucleus, becoming visible in images from immunostaining). The propagation of RSV and IAV for 48 h at an MOI of 0.01, along with the induction of STAT1 activity and its inhibition over time, is illustrated in Fig. S1 and S2, respectively, in the supplemental material.

We then analyzed the interferon-mediated activation of STAT1/2 during RSV or IAV infection and the consecutive induction of ISGs that are the primary correlates of the antiviral state: retinoic acid-inducible gene I (RIG-I) (viral RNA sensor) (51, 52), double-stranded RNA-activated protein kinase (PKR) (indirect translation inhibitor) (53), and 2′,5′-oligoadenylate synthetase 1 (OAS1) (direct activator of RNase L, the RNA eradicator) (54). We juxtaposed the results for A549 wild-type (WT) cells with those for the A549-derived cell lines in which we knocked out the IFN-α/β receptor IFNAR1, the heterodimeric IFN-λ receptor subunit IFNLR1, and both of these genes (Fig. 1D and E). We observed that infection with RSV or IAV leads to the robust activation (phosphorylation) of STAT1 and STAT2 in A549 WT cells as well as IFNLR1 KO cells. In IFNAR1-IFNLR1 dKO cells, there is no observable STAT1/2 phosphorylation, whereas in IFNAR1 KO cells, secreted type III IFNs (IFN-λ) lead to relatively weak STAT1/2 phosphorylation. The observed patterns of STAT activity translate directly to the patterns of accumulation of RIG-I, PKR, and OAS1, as well as STAT1 and STAT2, with the exception of IFNAR1-IFNLR1 dKO cells, in which we observed an increase in RIG-I and PKR after RSV infection, which is apparently independent of STAT1-STAT2 signaling. Cells devoid of the receptor for IFN-β, but not cells devoid of the receptor for IFN-λ, show higher levels of the RSV fusion glycoprotein, suggesting faster virus replication (Fig. 1D). Of note, the lack of either the IFN-β receptor or the IFN-λ receptor facilitates IAV replication despite the IFNLR1 KO having a minimal impact on STAT1/2 signaling and the accumulation of ISGs (Fig. 1E). Altogether, we conclude that despite the divergent efficacy of IFN-β induction by RSV and IAV, upon infection with any of these viruses, IFN-β is the main activator of STAT1 and STAT2 signaling. We did not analyze the impact of IFN-γ because the lack of STAT1 activation in IFNAR1 KO cells and IFNAR1-IFNLR1 dKO cells (Fig. 1D and E) suggests that this cytokine is not released by the studied cells and, as such, does not contribute to the inhibition of viral proliferation in our experimental setup.

### Prestimulation with IFN-β inhibits the propagation of RSV and IAV.

**FIG 2:**
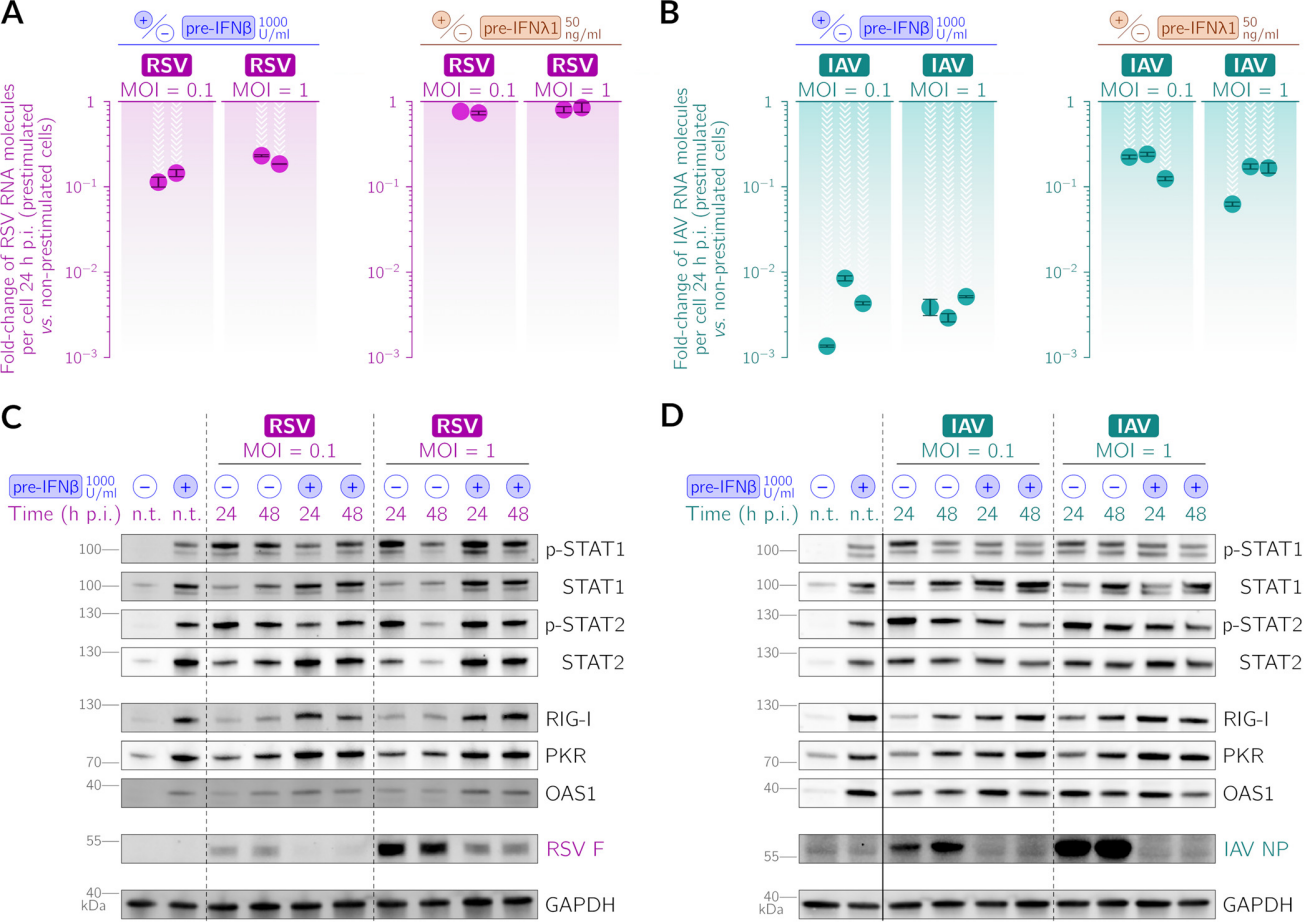
Prestimulation with IFN-β or IFN-λ1 inhibits the propagation of RSV and IAV in A549 WT cells. (A and B) Effect of prestimulation with IFN-β (1,000 U/mL) or IFN-λ1 (50 ng/mL) 24 h prior to infection on the relative abundance of viral RNA 24 h after RSV (A) and IAV (B) infection at MOIs of 0.1 and 1. Each filled circle corresponds to fold changes compared to nonprestimulated cells in a single experimental replicate (note the logarithmic scale). Error bars show the smallest and the largest ratios of nonprestimulated to IFN-prestimulated cells for all pairs of the respective technical replicates. (C and D) Effect of prestimulation with IFN-β (1,000 U/mL) 24 h prior to infection on the abundance and activation of STAT1 and STAT2 and the abundance of interferon-stimulated genes (RIG-I, PKR, and OAS1) and the RSV fusion glycoprotein (RSV F) (C) and IAV nucleoprotein (IAV NP) (D) at 24 and 48 h postinfection (p.i.) at MOIs of 0.1 and 1. n.t. indicates nontreated cells. Activated STATs p-STAT1 and p-STAT2 are STAT1 and STAT2 phosphorylated at Tyr701 and Tyr690, respectively.

To estimate the impact of priming with IFN-β and IFN-λ1 on the infectiousness and propagation of RSV and IAV, we quantified viral RNA molecules per cell and analyzed the abundance of viral proteins. We performed infection experiments without or with a preceding, day-long stimulation with IFN-β (1,000 U/mL) or IFN-λ1 (50 ng/mL). In Fig. 2, we juxtaposed the results of these two stimulation protocols. We found that 24 h after infection at an MOI of 0.1, prestimulation with IFN-β limits RSV progeny by nearly 1 order of magnitude, whereas IAV progeny are reduced by at least 2 orders of magnitude (cf. Fig. 2A and B). At an MOI of 1, when nearly all cells are infected, prestimulation with IFN-β limits RSV proliferation about 5-fold, while the relative reduction of IAV proliferation is as significant as that in the case of the lower MOI (cf. Fig. 2A and B). Prestimulation with IFN-β triggers an antiviral state manifested by the accumulation of ISGs, RIG-I, PKR, and OAS1, and STAT1/2. These proteins maintain their elevated levels at 48 h postinfection (Fig. 2C and D). Consequently, viral proteins are less abundant in IFN-β-prestimulated cells than in nonprestimulated cells. We summarize the results shown in Fig. 1 and 2 by stating that both IFN-β and IFN-λ secreted by infected cells are responsible for the confinement of the spread of IAV; however, only IFN-β appears to be capable of attenuating the propagation of RSV.

### Impact of RSV or IAV infection on STAT1 activation.

**FIG 3:**
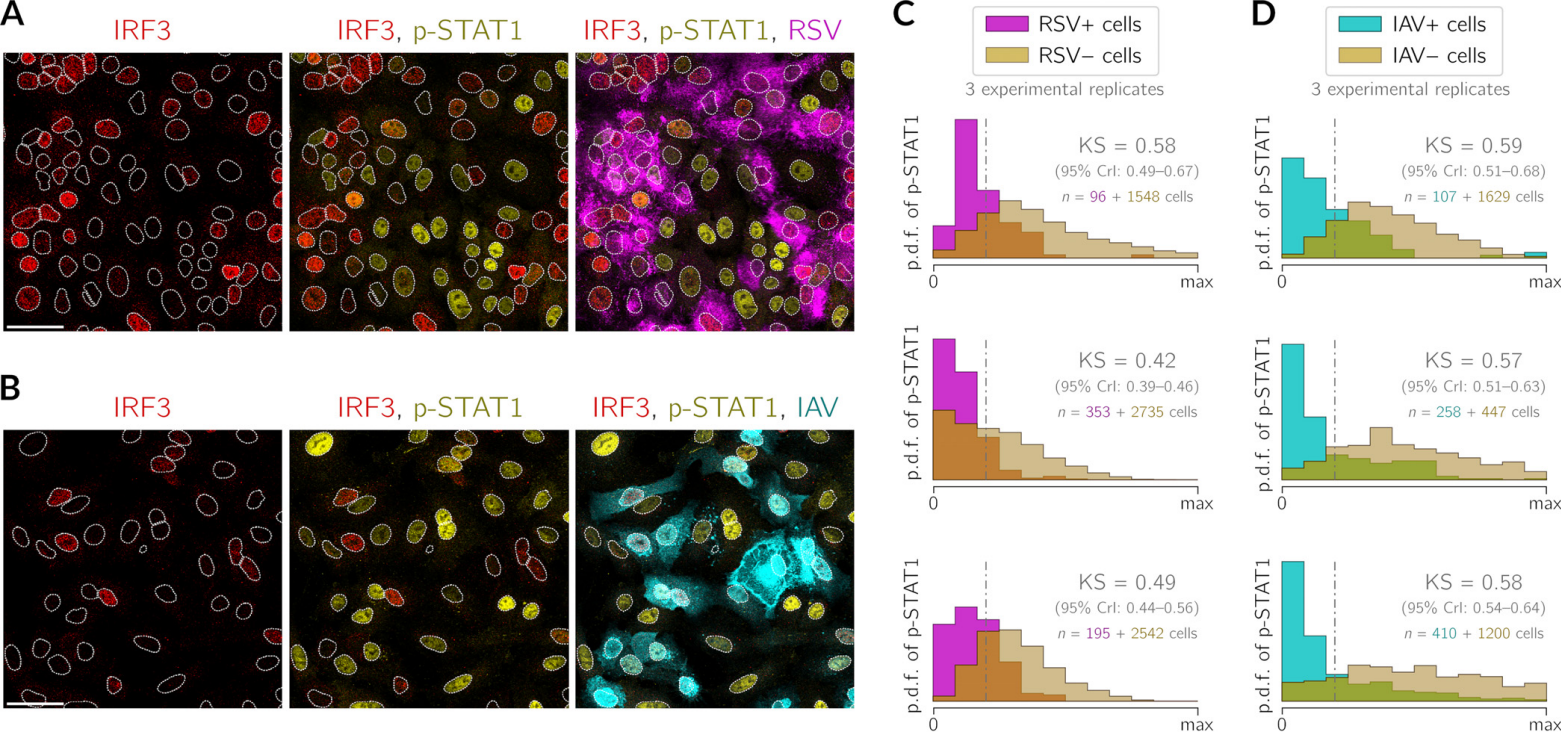
Impact of RSV or IAV infection on STAT1 activation. (A and B) A549 WT cells 24 h after infection with RSV (A) or IAV (B) at an MOI of 0.1. Channels with IRF3 (red), p-STAT1 (yellow), RSV (magenta, polyclonal anti-RSV antibody) (only in panel A), or IAV (cyan, anti-IAV nucleoprotein antibody) (only in panel B) are shown as incremental overlays. White dotted lines are nuclear outlines determined based on DAPI counterstaining (channel not shown). Bars, 50 μm. (C and D) Histograms showing the empirical probability density function (p.d.f.) of p-STAT1 24 h after infection with RSV (C) or IAV (D) at an MOI of 0.1 in cells classified as either expressing (+) or not expressing (−) the virus. For each pair of overlaid histograms, the Kolmogorov-Smirnov statistic is reported (with its credible interval [CrI]), and the corresponding maximally discriminating threshold is marked with a vertical dashed-dotted line. Each subpanel of panels C and D shows three experimental replicates separately, and the numbers of cells that were classified as virus positive and virus negative are given on the left- and right-hand sides of the plus sign, respectively.

To analyze at the single-cell level how infected cells induce an antiviral state in bystander cells, we performed infections at an MOI of 0.1, fixed the cells at 24 h postinfection, and immunostained them for active IRF3 (as evidence of the detection of viral RNA by cell), phosphorylated STAT1 (p-STAT1) (used as an indicator of the antiviral state), and viral proteins. In the two-channel overlays shown in Fig. 3A and B, it can be noticed that p-STAT1 is present in most bystander cells (at various levels), whereas the infected cells exhibit p-STAT1 only sporadically. This effect may indicate the ability of the viruses to suppress the response to IFN stimulation (46–50). We quantified this effect by looking directly at the presence of viral proteins as the definite manifestation of productive infection and at nuclear p-STAT1 as an indication of antiviral alertness. Based on the expression of RSV proteins or IAV nucleoprotein (NP), we classified cells as either virus positive or virus negative and collected histograms of the nuclear p-STAT1 intensity in each cell within the two groups. In Fig. 3C and D, we can see that the two distributions are indeed different for virus-positive and virus-negative cells, with on average higher STAT1 phosphorylation in virus-negative cells. This indicates that the antiviral state is more pronounced in bystander uninfected cells than in RSV-infected or IAV-infected cells at 24 h postinfection. To express the extent to which the two distributions are disjoint, we calculated the Kolmogorov-Smirnov (KS) statistic. The KS statistic attains values of 1 for fully disjointed distributions and 0 for exactly overlapping distributions. Across all experimental replicates, the KS values lie in the range of 0.42 to 0.58 (95% credible interval [CrI], 0.39 to 0.67) in the case of infection with RSV and in the range of 0.57 to 0.59 (95% CrI, 0.51 to 0.68) in the case of infection with IAV. See also Fig. S1 and S2 in the supplemental material, which document a decline in STAT1 phosphorylation in viral proteins-expresing cells.

### Effect of prestimulation with IFN-β or IFN-λ1 on IAV spread in A549 WT cells.

Thus far, we have used cell population techniques to show that IFN-β is a more potent inducer of the antiviral state than IFN-λ1 (Fig. 1), and as a consequence, in A549 cells, viral proliferation is more suppressed after prestimulation with IFN-β than after prestimulation with IFN-λ1 (Fig. 2A and B). Since RSV turned out to elicit a stronger IFN-β response than IAV, to characterize the potential interferon-mediated cross-protection between the two viruses, we decided to investigate specifically a dual-infection scheme in which the cells are first preinfected with RSV and then infected with IAV 24 h later.

**FIG 4:**
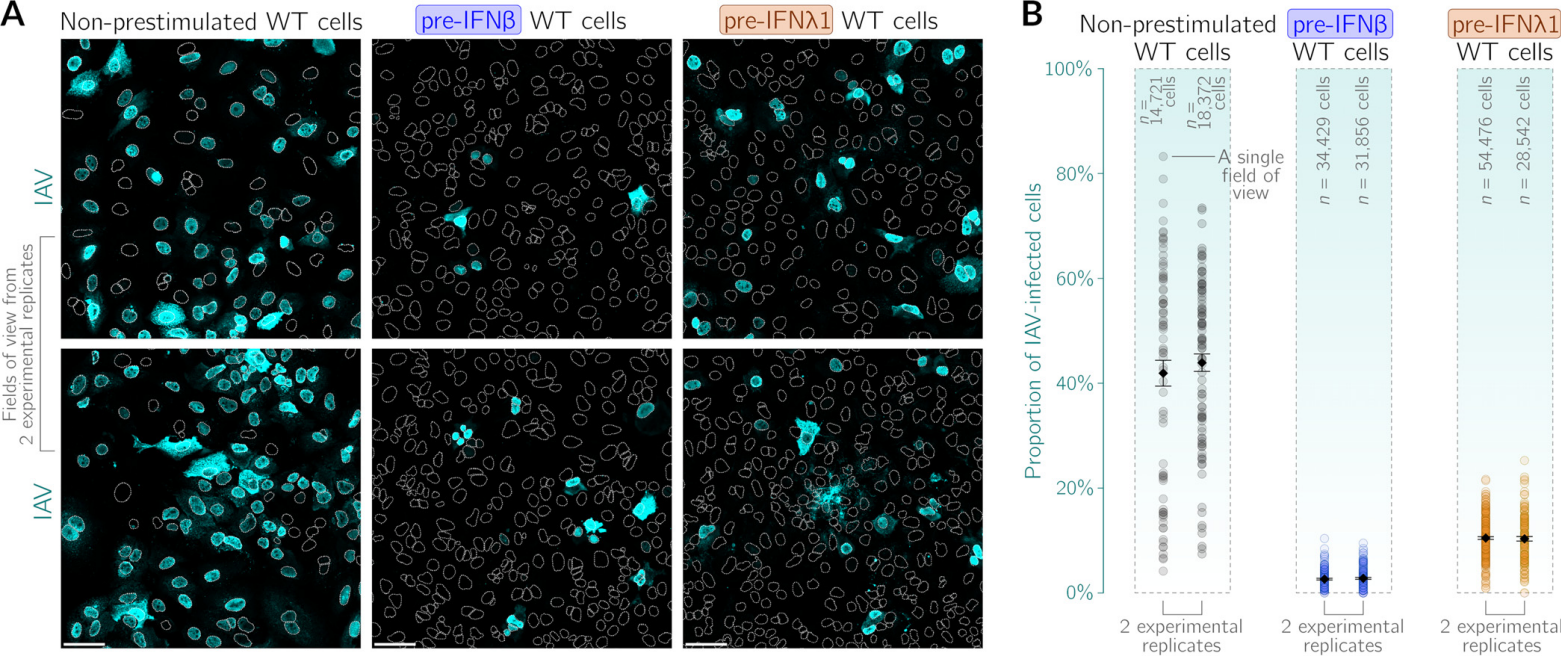
Effect of prestimulation with IFN-β or IFN-λ1 on IAV spread in A549 WT cells. Nonprestimulated A549 WT cells or A549 WT cells prestimulated with either IFN-β (100 U/mL) or IFN-λ1 (50 ng/mL) for 24 h were infected with IAV at an MOI of 1 and fixed at 24 h postinfection (i.e., 48 h since the beginning of prestimulation). Interferon-containing cell culture media were not displaced upon infection. (A) Representative fields of view showing nonprestimulated cells and cells prestimulated with either IFN-β or IFN-λ1 after infection with IAV (cyan, anti-IAV nucleoprotein antibody). White dotted lines are nuclear outlines determined based on DAPI counterstaining (channel not shown). For each pretreatment condition, sample images from two experimental replicates are shown. Bars, 50 μm. (B) Proportions of productively IAV-infected cells among nonprestimulated cells and cells prestimulated with either IFN-β or IFN-λ1 based on nucleoprotein expression. Data from two independent experiments are shown separately; each circle corresponds to a single field of view. The fields of view do not overlap. Black diamonds and error bars denote mean proportions ± standard errors of the means (SEM). The numbers of cells analyzed in all fields of view from each experiment are given above the respective data series.

To gain insight into the interferon-mediated interaction at the single-cell level, we quantified the protective effect of IFN-β or IFN-λ1 in the context of IAV infection based on imaging data (Fig. 4A). Figure 4B shows the proportions of cells expressing viral nucleoprotein 24 h after treatment with IAV at an MOI of 1 in each field of view in the cases of nonprestimulated A549 WT cells and A549 WT cells that were prestimulated with either IFN-β or IFN-λ1. Based on image quantifications for two experimental replicates, we found that 40 to 45% of nonprestimulated cells express nucleoprotein 24 h after infection. This proportion drops to about 3% after prestimulation with IFN-β and to about 10% after prestimulation with IFN-λ1. These proportions provide additional evidence for the strong attenuation of IAV spread conferred by IFNs in A549 cells.

### RSV preinfection inhibits IAV infection in an interferon-dependent manner.

In the course of viral infection, there are cells that are initially infected by the primary virus and bystander cells, which, in response to IFN secreted by the initially infected cells, may develop an antiviral state and become partially protected from secondary infections. Our previous experiments with IFN prestimulation and a high IAV MOI allowed us to characterize the response of the cells that had uniformly developed the antiviral state and were then challenged with the virus, which mimics only one subpopulation of the cells that appears during ongoing infection. Since in our dual-infection scheme, we wanted to apply the second virus to a cell population that consists of the subpopulation of the preinfected and IFN-producing cells and the subpopulation of not (yet)-infected but IFN-stimulated cells, we primed cells with the first virus, RSV, at an MOI of 0.1, and after 30 h, we applied the second virus, IAV, at an MOI of 1. To corroborate and discern the relative importance of IFN-β or IFN-λ in the native RSV-elicited mixture, we additionally used A549 IFNAR1 KO, IFNLR1 KO, and IFNAR1-IFNLR1 dKO cell lines.

**FIG 5:**
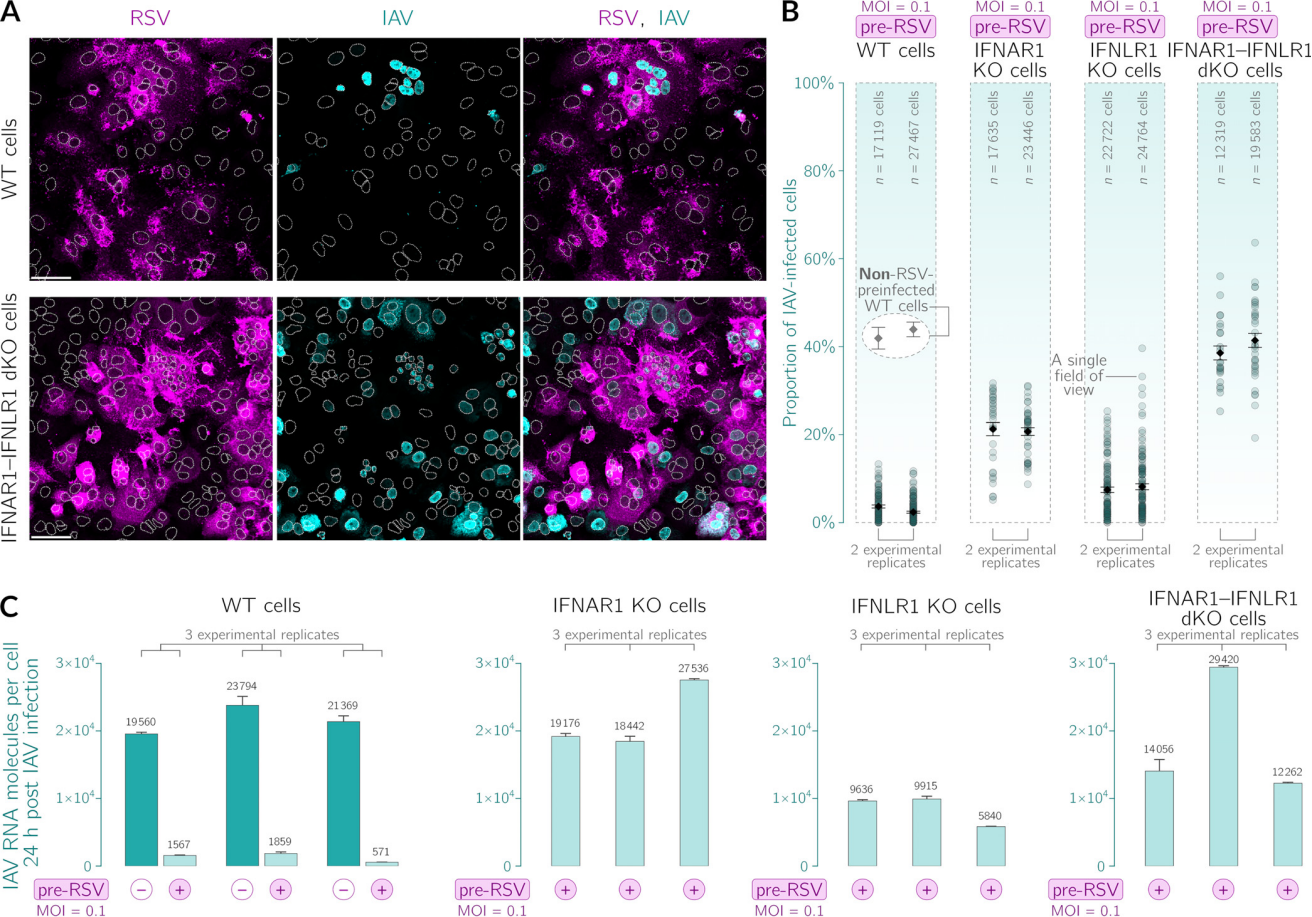
RSV preinfection inhibits IAV infection in an interferon-dependent manner in A549 cells. Analyzed are infections in four A549 cell lines: WT, IFNAR1 KO, IFNLR1 KO, and IFNAR1-IFNLR1 double KO (dKO). Nonpreinfected cells and cells preinfected with RSV at an MOI of 0.1 for 30 h were infected with IAV at an MOI of 1 and cultured subsequently for 24 h. Cell culture medium was not displaced upon IAV infection. (A) Representative fields of view showing WT and IFNAR1-IFNLR1 dKO cells preinfected with RSV (magenta, polyclonal anti-RSV antibody) after infection with IAV (cyan, anti-IAV nucleoprotein antibody). White dotted lines are nuclear outlines determined based on DAPI counterstaining (channel not shown). Bars, 50 μm. (B) Proportions of IAV-infected cells in an RSV-preinfected cell population. Data from two independent experiments are shown separately; each circle corresponds to a single field of view. Black diamonds and error bars denote mean proportions ± SEM. The two encircled gray diamonds above the data points for WT cells are the mean proportions of IAV-infected WT cells that were not subjected to preinfection with RSV (nonprestimulated WT cells in Fig. 4B), shown here again as a reference. (C) Impact of preinfection with RSV on the number of IAV RNA molecules per cell in all four considered cell lines. Cells were infected with RSV and then IAV 30 h later. dPCR was performed for cells collected 24 h after IAV (i.e., 54 h after RSV) infection. Error bars are SD from technical replicates.

In Fig. 5A and B, we show an imaging-based single-cell-level analysis of the cell population after dual infection. After preinfection with RSV, the proportion of WT A549 cells infected with the second virus, IAV, is reduced by an order of magnitude with respect to nonpreinfected cells (cf. nonprestimulated WT cells in Fig. 4B and RSV-primed WT cells in Fig. 5B, which gives a 10-fold reduction of IAV nucleoprotein-expressing cells, from ~40% to ~4%). The protective effect conferred by RSV priming is significantly weaker in IFNAR1 KO cells incapable of responding to IFN-β (reduction from ~40% to ~20%) and only slightly weaker in IFNLR1 KO cells incapable of responding to IFN-λ (reduction from ~40% to ~10%). The priming effect is virtually nonexistent in cells devoid of receptors for both IFN-β and IFN-λ. According to the digital PCR (dPCR) measurements shown in Fig. 5C, the amount of IAV RNA in a population of RSV-preinfected WT cells is 10 to 20 times smaller than that in a nonpreinfected population of WT cells. Additionally, in populations of RSV-preinfected cells from three KO cell lines, the amount of IAV RNA is larger than that in the WT cell population, and the ratios of IAV RNA expression between these lines are similar to the ratios for IAV-infected cells shown in Fig. 5B. Since IFNs block IAV at the levels of the replication of viral RNA and the synthesis of viral proteins, the accumulating effect at the level of viral proteins may be expected to be more pronounced.

**FIG 6:**
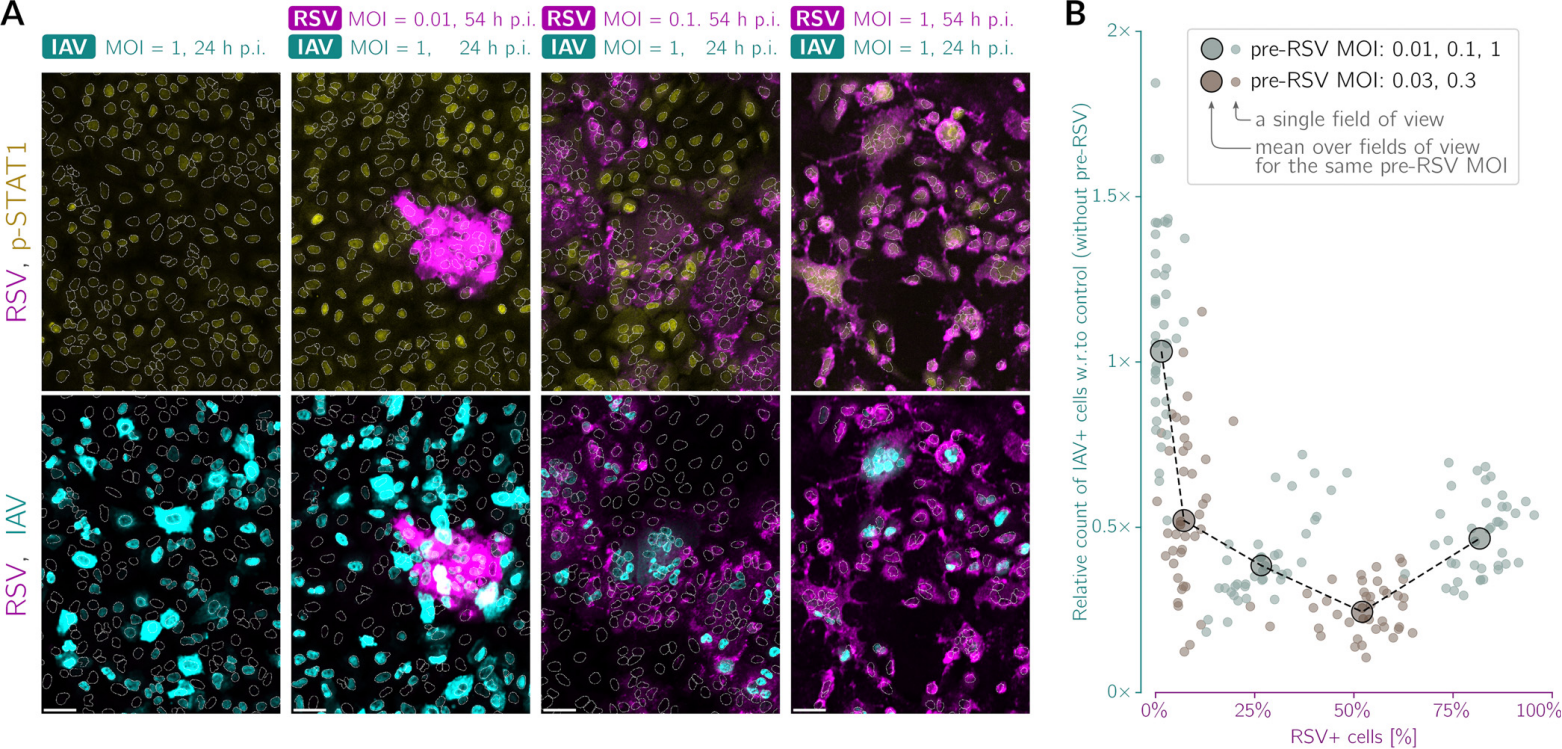
RSV preinfection inhibits IAV infection in an RSV MOI-dependent manner. (A) Representative fields of view showing A549 WT cells preinfected with RSV at different MOIs (0.01, 0.03, 0.1, 0.3, and 1) for 30 h and then infected with IAV for an additional 24 h. (Top) Overlays of RSV (magenta, polyclonal anti-RSV antibody) and p-STAT1 (yellow); (bottom) overlays of RSV (magenta, polyclonal anti-RSV antibody) and IAV (cyan, anti-IAV nucleoprotein antibody). White dotted lines are nuclear outlines determined based on DAPI counterstaining (channel not shown). Bars, 50 μm. (B) Proportions of the counts of IAV-infected cells after preinfection with RSV to the counts of IAV-infected cells without preinfection as a function of the percentage of cells expressing RSV proteins. Small circles correspond to individual fields of view, whereas large circles represent values averaged over all fields of view for a given MOI of RSV preinfection. Data are from two representative experiments; the RSV stock used in these experiments (performed in manuscript revision) was different from that in the experiments in Fig. 1 to 3, 5, and 7. w.r., with respect.

In Fig. 6A and B, we demonstrate that the protective effect conferred by RSV priming depends on the RSV MOI. The strongest protection is achieved for an intermediate RSV MOI of ~0.1 to 0.3, whereas for an RSV MOI of ~1, protection is slightly weaker, and for an RSV MOI of ~0.01, it is much weaker (Fig. 6B). At the lowest considered RSV MOI, a small number of cells secrete IFNs, which is likely insufficient to evoke a pervasive antiviral state in bystander cells. We note that varying the pre-RSV MOI is somewhat similar to changing the time interval between subsequent infections because longer intervals permit more secondary infections with the priming virus and greater accumulation of interferons.

Overall, this demonstrates that preinfection with RSV protects A549 cells from infection with IAV in an interferon-dependent manner. The protective effect may be attributed nearly entirely to paracrine stimulation with both IFN-β and IFN-λ, with a major contribution from IFN-β.

### IAV preferentially infects cells that express RSV proteins.

**FIG 7:**
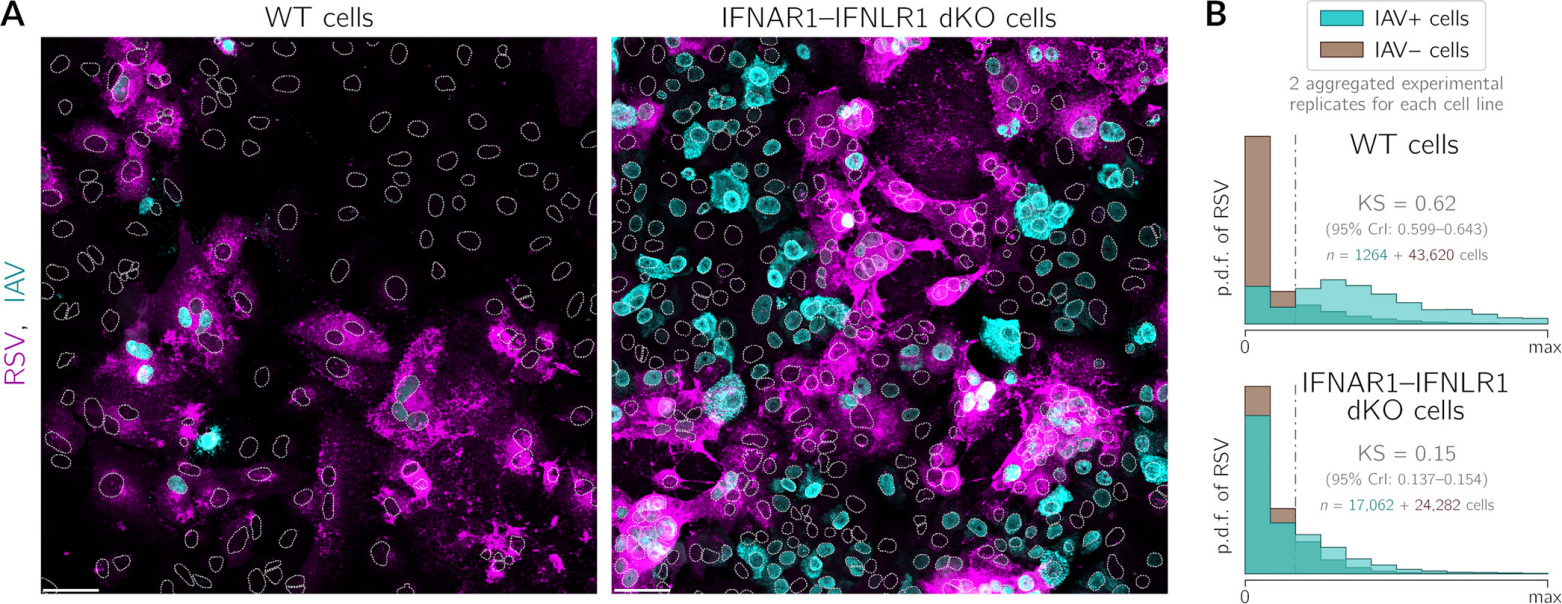
IAV preferentially infects A549 WT cells expressing RSV proteins, but this effect is not observed in A549 IFNAR1-IFNLR1 dKO cells. A549 WT and A549 IFNAR1-IFNLR1 dKO cells were preinfected with RSV (MOI = 0.1) 30 h before infection with IAV (MOI = 1) and fixed 24 h after infection with IAV. (A) Representative fields of view showing WT cells and IFNAR1-IFNLR1 dKO cells preinfected with RSV (magenta, polyclonal anti-RSV antibody) after subsequent IAV (cyan, anti-IAV nucleoprotein antibody) infection (overlays). White dotted lines are nuclear outlines determined based on DAPI counterstaining (channel not shown). Bars, 50 μm. (B) Histograms of RSV intensity (p.d.f., probability density function) in cells classified as either infected (cyan) or not infected (brown) with IAV in A549 WT cells and A549 IFNAR1-IFNLR1 dKO cells. Histograms show aggregated data from two experimental replicates. The numbers of cells that were classified as virus positive and virus negative are given on the left- and right-hand sides of the plus sign, respectively.

Visual inspection of WT cells suggested that IAV nucleoprotein is preferentially expressed in cells with apparent expression of RSV proteins (Fig. 7A). To quantitatively verify this hypothesis, as shown in Fig. 3D, we classified cells as virus positive and virus negative. We decided to use the measured level of IAV NP as the binary criterion because of its mostly nuclear location, which facilitates image analysis. We then collected histograms of the intensities of RSV proteins in the cells belonging to these two groups (Fig. 7B). Clearly, in A549 WT cells, the IAV-positive (IAV+) cells are associated with a higher abundance of RSV proteins than IAV-negative (IAV−) cells (the KS value of 0.62 [95% CrI, 0.599 to 0.643] indicates good discernibility). This effect is much weaker in IFNAR1-IFNLR1 dKO cells, which cannot respond to IFN-β or IFN-λ (the KS value of 0.15 [95% CrI, 0.137 to 0.154] indicates that the two distributions are nearly overlapping). This clearly shows that RSV-IAV exclusion is not realized through direct competition for a shared ecological niche, a single cell, but rather is achieved with the involvement of interferon signaling.

Based on the distributions shown in Fig. 7B, we introduced a threshold at which two distributions are best separable (vertical dotted-dashed line), allowing us to stratify cells into RSV-positive and RSV-negative ones. We then found in experiments with WT cells that among the RSV-positive cells (which constituted 20% of all cells), 11% were IAV positive, whereas among the RSV-negative cells (which constituted 80% of all cells), <1% were IAV positive. In experiments with dKO cells, among the RSV-positive dKO cells (which constituted 25% of all cells), 56% were IAV positive, and among the RSV-negative cells (which constituted 75% of all cells), as much as 37% were IAV positive.

**FIG 8:**
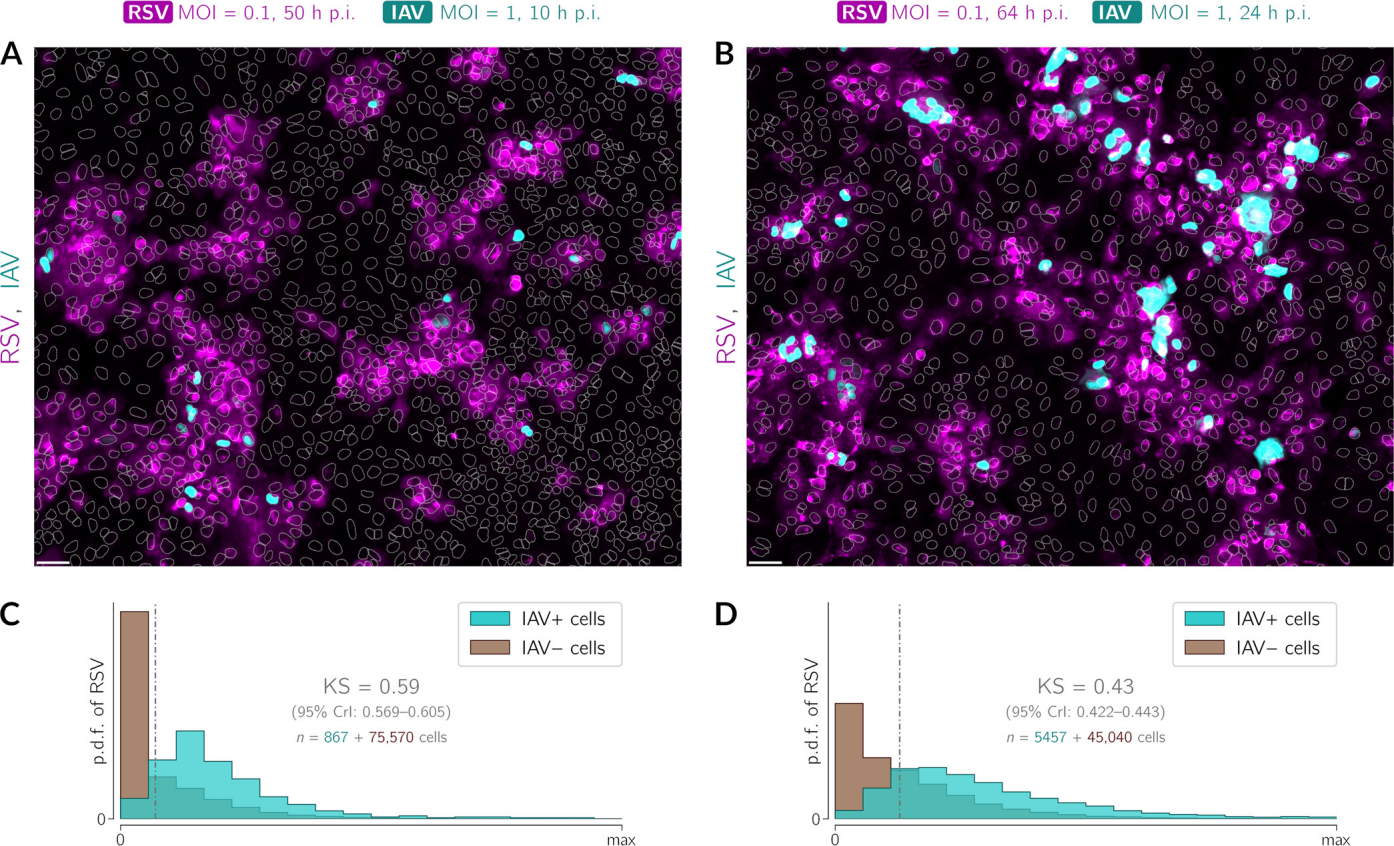
IAV’s preference to infect RSV-preinfected cells is maintained over time. A549 WT cells were preinfected with RSV (MOI = 0.1) 40 h before infection with IAV (MOI = 1) and fixed 10 or 24 h after infection with IAV. (A and B) Representative fields of view showing cells preinfected with RSV (magenta, polyclonal anti-RSV antibody) after subsequent IAV (cyan, anti-IAV nucleoprotein antibody) infection (overlays) 10 h (A) and 24 h (B) after infection with IAV. White dotted lines are nuclear outlines determined based on DAPI counterstaining (channel not shown). Bars, 50 μm. (C and D) Histograms of RSV intensity (p.d.f., probability density function) in cells classified as either infected (cyan) or not infected (brown) with IAV. The numbers of cells that were classified as virus positive and virus negative are given on the left- and right-hand sides of the plus sign, respectively. Data are from two representative experiments; the RSV stock used in these experiments (performed in manuscript revision) was different from that in the experiments in Fig. 1 to 3, 5, and 7.

To demonstrate that the RSV-and-IAV coinfections observed at the single-cell level are due mainly to IAV infections of cells that already express RSV proteins, we performed an experiment in which 40 h after infection with RSV (at an MOI of 0.1), cells are infected with IAV for either 10 or 24 h (Fig. 8). Using this protocol, it is less probable that a cell that has not been infected during the first 40 h becomes infected jointly with two viruses in the last 10 h of the experiment. Immunostaining images (Fig. 8A and C) clearly show that the localization of IAV-infected cells recapitulates the spatial pattern of RSV-infected cell clusters, and the cells infected with IAV have a higher chance of being infected with RSV (and vice versa). This effect is even stronger 10 h than 24 h after infection with IAV (Fig. 8B and D).

As in the case of Fig. 7B, we stratified cells into RSV-positive and RSV-negative cells. We then found that 10 h after infection with IAV (Fig. 8C), among the RSV-positive cells (which constituted 34% of all cells), 3% were IAV positive, whereas among the RSV-negative WT cells (which constituted 66% of all cells), about 0.1% were IAV positive. We also found that 24 h after infection with IAV (Fig. 8D), 46% of all cells were RSV positive, and among these cells, 20% were IAV positive; among the RSV-negative cells (54%), about 3% were IAV positive.

## DISCUSSION

To replicate, an invading pathogen has to evade or compromise innate immunity. When challenged by a virus, the innate immune system acts to impede pathogen replication before a more specific adaptive response is evoked. In the context of sequential viral coinfections, the priming virus both breaks through the innate protection in infected cells and indirectly, via interferon secreted by these cells, triggers a protective antiviral state in bystander cells. These two antagonistic processes determine the fate of both the primary and secondary infections at the cell-population level.

To obtain insight into this interplay at the single-cell level, we studied how upon a priming infection with RSV, individual cells become either cross-protected or, conversely, more vulnerable to the second virus, IAV. The choice of these two pathogens has been motivated by the fact that RSV and influenza viruses show a relatively high coincidence (55). The particular order of infections that we focused on results from our observations that (i) RSV induces higher levels of IFN-β production than IAV, (ii) IFN-β-stimulated cells are more resistant to infection with IAV than to infection with RSV, and (iii) IAV is sensitive to IFN-λ, and this sensitivity does not rely solely on STAT1/2 signaling. We thus inferred that the potential cross-protection may be more pronounced in the RSV-then-IAV protocol, which is consistent with a comparison of RSV-then-IAV and IAV-then-RSV coinfections in a mouse model showing that IAV-then-RSV coinfection is associated with higher IAV loads and mouse mortality rates (56; see also reference 57).

We found that IFN-β is the main inducer of the STAT1/2-associated antiviral state in RSV-infected A549 cells and that both IFN-β and IFN-λ are simultaneously necessary for building maximum protection against subsequent infection with IAV. A synergistic effect of type I and type III interferons, consistent with our findings, has been previously reported in mice by Mordstein et al. (58). In our coinfection experiments, we infected epithelial alveolar cells with RSV at an MOI of 0.1 to readily observe their stratification into compromised infected cells and reinforced bystander cells. Immunostaining revealed that preinfection with RSV partitions the cell population into a subpopulation susceptible to subsequent infection with IAV and an IAV-proof subpopulation. This dynamic functional differentiation of cells may be amplified *in vivo* by the differential propensities of specific cell types to permit or restrict viral replication, produce interferons, and build up an antiviral state (59). We found that the susceptible cells turned out to be predominantly those already compromised and efficiently expressing the priming virus. This means that after preinfection, the population of cells susceptible to a secondary infectant is limited largely to those yielding preinfectant progeny, the proportion of which depends on the multiplicity of preinfection and the time interval between infections. An optimal cross-protective effect may thus ensue when the proportion of cells preinfected with RSV is relatively low yet sufficient to trigger a pervasive antiviral state in bystander cells. In the case of massive infection with RSV, when most cells are simultaneously compromised by the virus, interferon-mediated protection likely does not come into effect. The protective effect of RSV infection may increase with the yield of DVGs, which, due to the lack of nonstructural proteins, may both more efficiently trigger the synthesis of interferons and leave STAT signaling unscathed.

The natural question is to what extent the above-described cross-protection mechanism reduces the incidence of coinfections or the severity of the disease featured by secondary infection *in vivo*. Answering this question is especially important but appears complex in the context of secondary SARS-CoV-2 infections. An early study by Ziegler et al. (60) showed that the receptor for SARS-CoV-2, ACE2, is a protein encoded by an ISG, but later, Onabajo et al. (61) identified a transcriptionally independent truncated isoform of *ACE2*, *deltaACE2*, not *ACE2*, as an ISG. Since deltaACE2 may not bind the SARS-CoV-2 spike protein, the latter study suggests that interferon signaling is not hijacked by this virus to enhance its proliferation. Several studies suggested the beneficial roles of type I and type III interferons at the early stage of infection (62). It is thus possible that interferons secreted in response to primary infection exhibit a protective role against secondary infection with SARS-CoV-2 (see also reference 63).

At the clinical level, one may expect that the occurrence of coinfections may result from defective work of the immune system (which aggravates the course of the disease and also imaginably cannot mediate antagonistic viral interactions). Although this effect cannot be excluded, and coinfection data should be treated with caution, the impact of coinfections on disease severity is unclear. Most respiratory viral coinfections, detected usually in a small proportion of patients, appear not to increase the severity of clinical outcomes (64–67), with two noteworthy exceptions, coinfection by IAV and influenza B virus (IBV) (55) and coinfections with RSV in pediatric patients (68). It should be noted that the interactions of two respiratory viruses within the host can hardly be translated to their coincidence or avoidance in human populations. This is because at the population level, there are two additional and antagonistic effects: on the one hand, the viral coincidence of contagious diseases can be seasonal (the effective contact rate is modulated by weather and behavioral patterns); on the other hand, infected and ill individuals change their behavior to avoid contact. A significant behavioral reduction of the contact rate may result from an outbreak of an epidemic of a threatening virus, decreasing the risk of the propagation of other viruses, as observed during the coronavirus disease 2019 (COVID-19) pandemic in 2020 (69, 70).

In conclusion, our study demonstrates that infection with RSV at a low MOI protects alveolar epithelial cells against infection with IAV. Whereas RSV-infected cells are more vulnerable to infection with IAV, priming with RSV indirectly protects bystander noninfected cells from IAV. This cross-protection mechanism relies on the induction of and paracrine stimulation with both type I and type III interferons.

## MATERIALS AND METHODS

### Cell lines and cultures.

The A549 and MDCK cell lines were purchased from the ATCC. A549 cells were cultured in F-12K basal medium supplemented with 10% fetal bovine serum (FBS) and a penicillin-streptomycin antibiotic solution. MDCK cells and HEK-293T cells were maintained in Dulbecco’s modified Eagle’s medium (DMEM) supplemented with 10% FBS and a penicillin-streptomycin antibiotic solution. All cell lines were cultured under standard conditions (37°C with 5% CO_2_) and kept in monolayers up to 90% confluence. The IFNAR1, IFNLR1, and IFNAR1-IFNLR1 knockouts based on the A549 cell line were engendered using the CRISPR lentivirus system (Abm). To obtain A549-based knockouts, HEK-293T cells were cotransfected with commercially available plasmids encoding Cas9 nuclease (catalog number K002; Abm), lentivirus packaging particles (catalog number LV053; Abm), and single guide RNA (sgRNA) targeted against IFNAR1 (catalog number 2426411; Abm) or IFNLR1 (catalog number 2487611; Abm). The IFNAR1-IFNLR1 double mutant was created based on IFNAR1 KO cells. Two days after transfection, the media with lentiviral particles were collected, enriched with 8 μg/mL Polybrene, and filtered through 0.45-μm syringe filters. Next, each lentiviral supernatant was used to transduce A549 wild-type cells, which were subcultured at low confluence (seeding density of 5 × 10^4^ cells per 30-mm dish). After another 2 days, A549 cells were subjected to selection with 800 μg/mL of G418 for 10 days and seeded into a 96-well plate to obtain single-cell colonies. Clones were validated by Western blotting of p-STAT1 and p-STAT2 in response to IFN-β or IFN-λ1 (see Fig. S3 in the supplemental material). Additionally, the KO clones selected for further experiments were verified by sequencing.

### Virus amplification and isolation.

Respiratory syncytial virus strain A2 and influenza A virus H1N1 strain A/PR/8/34 were purchased from the ATCC and amplified in HeLa and MDCK cells, respectively. Cells were seeded into 225-cm^2^ tissue culture flasks (Falcon) and cultured as described above for 2 to 3 days until they reached 90% confluence. On the day of infection, the following virus growth medium was prepared: DMEM with 2% FBS for RSV or basal minimal essential medium (MEM) with 0.3% bovine serum albumin (BSA) and 1 μg/mL of L-1-tosylamido-2-phenylethyl chloromethyl ketone (TPCK-trypsin) for IAV. The dilutions of virus were prepared in an appropriate medium, with target MOIs of around 0.01 for RSV and 0.05 for IAV. Culture media were removed, and cells were washed once with phosphate-buffered saline (PBS) and overlaid with 10 mL of the inoculum. Virus was allowed to adsorb to cells for 2 h at 37°C, with occasional stirring. Next, additional virus growth medium was added to a total volume of 40 mL per flask. Infected cells were cultured at 37°C until the development of cytopathic effects could be observed in at least 80% of the cells (typically at around 3 days for IAV and 5 days for RSV). Virus-containing culture fluid was then collected and clarified by centrifugation at 3,000 × *g* at 4°C for 20 min. Next, virus particles were precipitated by adding 50% (wt/vol) polyethylene glycol 6000 (PEG 6000) (Sigma-Aldrich) in NT buffer (150 mM NaCl, 50 mM Tris-HCl [pH 7.5]) to a final concentration of 10% and stirring the mixture gently at 4°C for 90 min. Virus was centrifuged at 3,250 × *g* at 4°C for 20 min and recentrifuged after the removal of the supernatant to remove the remaining fluid. The pellet was suspended in 1 mL of NT buffer or in 20% sucrose in NT buffer in the case of RSV, aliquoted, and stored at −80°C.

### Virus quantification.

The virus concentration in the collected samples was quantified using an immunofluorescence protocol. HeLa or MDCK cells were seeded onto microscopic coverslips and cultured upon reaching 90 to 100% confluence. Serial dilutions of virus samples were made in virus growth medium in a range of 10^−3^ to 10^−6^. After washing with PBS, cells were overlaid in duplicates with diluted virus, which was allowed to adhere for 2 h, with occasional stirring. Afterward, the virus-containing medium was removed, and cells were overlaid with fresh virus growth medium and cultured for 16 h (for IAV) or 24 h (for RSV). Next, cells were washed with PBS and fixed with 4% formaldehyde for 20 min at room temperature. Cells were stained using a standard immunofluorescence protocol with anti-RSV fusion glycoprotein antibody (catalog number ab43812; Abcam) or anti-influenza A virus nucleoprotein antibody (clone C43) (catalog number ab128193; Abcam). Cells containing stained viral proteins were counted using a Leica SP5 confocal microscope. The virus concentration was calculated using the following formula: (average number of infected cells)/(dilution factor × volume containing virus added) = infectious particles/mL. For a given MOI, we observe fewer cells expressing viral proteins in the A549 than in the HeLa and MDCK lines, which are known to be permissive for RSV and IAV infections, respectively.

### Compounds and stimulation protocols.

Human IFN-β1A and IFN-λ1 were purchased from Thermo Fisher Scientific (catalog numbers PHC4244 and 34-8298-64, respectively) and prepared according to the manufacturer’s instructions. For cell stimulation, interferons were further diluted to the desired concentrations in F-12K medium supplemented with 2% FBS. The decreased FBS content was used to prevent the inhibition of viral attachment and entry at the second stage of the experiments.

For interferon-virus experiments, the cell culture media were exchanged for interferon-containing or control media at time zero and were not removed afterward until the end of the experiment. Appropriately diluted virus was added in small volumes (less than 10 μL) directly into the wells. The even distribution of virus across the cell population was aided by the intermittent rocking of the plate for 2 h.

Similarly, for coinfection experiments, F-12K–2% FBS medium containing the first virus (RSV) was added to the cells at the beginning of the experiment, while the dilutions of the second virus (IAV) were added directly to the wells at a later time point and distributed by rocking.

For intracellular IFN-β visualization, a brefeldin A solution (BD Biosciences) was added to cells 2 h prior to fixation.

### Antibodies.

**(i) Antibodies for Western blotting.** Primary antibodies for Western blotting were anti-phospho-STAT1 (Tyr701) (clone 58D6) (catalog number 9167; Cell Signaling Technologies) (1:1,000), anti-phospho-STAT2 (Tyr690) (clone D3P2P) (catalog number 88410; Cell Signaling Technologies) (1:1,000), anti-RIG-I (clone D14G6) (catalog number 3743; Cell Signaling Technologies) (1:1,000), anti-STAT1 (catalog number 610116; BD Biosciences) (1:1,000), anti-STAT2 (catalog number PAF-ST2; R&D Systems) (1:1,000), anti-PKR (clone B-10) (catalog number sc-6282; Santa Cruz Biotechnology) (1:1,000), anti-OAS1 (clone F-3) (catalog number sc-374656; Santa Cruz Biotechnology) (1:1,000), anti-influenza A virus nucleoprotein (clone C43) (catalog number ab128193; Abcam) (1:1,000), anti-respiratory syncytial virus (clone 2F7) (catalog number ab43812; Abcam) (1:1,000), and hFAB rhodamine anti-glyceraldehyde-3-phosphate dehydrogenase (GAPDH) (catalog number 12004168; Bio-Rad) (1:10,000).

Secondary antibodies for Western blotting were DyLight 800 4× PEG-conjugated goat anti-rabbit IgG(H+L) (catalog number SA5-35571; Thermo Fisher Scientific) (1:10,000), DyLight 800 4× PEG-conjugated goat anti-mouse IgG(H+L) (catalog number SA5-35521; Thermo Fisher Scientific) (1:10,000), StarBright Blue 700-conjugated goat anti-mouse IgG (catalog number 12004159; Bio-Rad) (1:10,000), and horseradish peroxidase (HRP)-conjugated rabbit anti-goat immunoglobulins (catalog number P0449; Agilent) (1:10,000).

**(ii) Antibodies for immunostaining.** Primary antibodies for immunostaining were anti-phospho-STAT1 (Tyr701) (clone 58D6) (catalog number 9167; Cell Signaling Technologies) (1:1,000), anti-IRF3 (clone D-3) (catalog number sc-376455; Santa Cruz Biotechnology) (1:500), anti-respiratory syncytial virus (catalog number ab20745; Abcam) (1:1,000), anti-influenza A virus (catalog number ab20841; Abcam) (1:1,000), anti-influenza A virus nucleoprotein (clone C43) (catalog number ab128193; Abcam) (1:1,000), and anti-human interferon beta (catalog number MAB8142; R&D Systems).

Secondary antibodies for immunostaining were Alexa Fluor 488-conjugated donkey anti-rabbit IgG(H+L) (catalog number A-21206; Thermo Fisher Scientific) (1:1,000), Alexa Fluor 555-conjugated donkey anti-mouse IgG(H+L) (catalog number A-31570; Thermo Fisher Scientific) (1:1,000), and Alexa Fluor 633-conjugated donkey anti-goat IgG(H+L) (catalog number A-21082; Thermo Fisher Scientific) (1:1,000).

### ELISA.

For measuring IFN-β production, cells were seeded onto 96-well plates (Falcon) at a density of 20,000 cells per well and infected with RSV or IAV as described above. The culture medium (total of 200 μL) from infected cells was collected at the designated time points and stored at −20°C until further analysis. IFN-β levels were estimated using the VeriKine mouse IFN-β enzyme-linked immunosorbent assay (ELISA) kit (PBL Assay Science). Standards and diluted samples in duplicates or triplicates were added to a precoated plate included in the kit and incubated for 1 h. After subsequent washing, an antibody solution was prepared and added to wells for another 1 h, followed by another wash and 1 h of incubation with an HRP solution. Finally, a TMB substrate solution was added to the wells, and the developing color reaction was stopped after 15 min with the addition of a stop solution. The optical densities of the samples after the resulting color development were determined using a Multiskan Go plate reader (Thermo Fisher Scientific) set to 450 nm, with wavelength correction at 570 nm. IFN-β concentrations were obtained based on the standard curve (4-parameter logistic function fitted to 7 data points from the standards).

### Digital PCR.

For gene expression experiments, cells were seeded onto 24-well plates at a density of 100,000 cells per well. RNA from virus-infected cells was isolated using a PureLink RNA minikit (Thermo Fisher Scientific) according to the manufacturer’s instructions: cells were harvested and vortexed in lysis buffer with 2-mercaptoethanol and then vortexed again with 1 volume of 70% ethanol. Upon transfer to the spin cartridge, the cellular RNA content was bound to the column, washed with the appropriate buffers, and eluted, all by centrifugation at 12,000 × *g*. The eluted RNA in RNase-free water was used immediately for reverse transcription (RT) or stored for later use at −80°C. The concentration and quality of the isolated total RNA were determined by measuring the UV absorbance at 260 nm and 280 nm using a Multiskan Go spectrophotometer (Thermo Fisher Scientific). Approximately 0.5 μg of RNA was used as a template for reverse transcription, performed using a high-capacity cDNA reverse transcription kit (Thermo Fisher Scientific): the diluted RNA samples were mixed 1:1 with freshly prepared master mix containing RT buffer, RT random primers, deoxynucleoside triphosphate (dNTP) mix, MultiScribe reverse transcriptase, and an RNase inhibitor. The reaction was performed in a Mastercycler gradient thermal cycler (Eppendorf) under the following conditions: 10 min at 25°C, 120 min at 37°C, and 5 min at 85°C.

Measurements of the viral RNA copy number were then performed using the QuantStudio 3D system (Thermo Fisher Scientific) and TaqMan Vi99990011_po and Vi99990014_po gene expression assays for the quantification of IAV and RSV RNAs, respectively. Appropriately diluted samples were mixed with the reaction master mix and the chosen assay mix, loaded onto the digital PCR (dPCR) chips in duplicates, and thermocycled using a ProFlex flat-block thermal cycler. Subsequently, chips were analyzed with a QuantStudio 3D digital PCR instrument. The chip quality check and final data analysis were performed using QuantStudio 3D AnalysisSuite software.

### Western blotting.

Cells at the indicated time points were washed twice with PBS, lysed in Laemmli sample buffer containing dithiothreitol (DTT), and boiled at 95°C for 10 min. Equal amounts of each protein sample were separated on 4 to 16% Mini-Protean TGX stain-free precast gels using the Mini-Protean Tetra cell electrophoresis system (Bio-Rad). Upon the completion of electrophoresis, proteins were transferred to a nitrocellulose membrane using wet electrotransfer in the Mini-Protean apparatus (400 mA for 50 min). The membrane was rinsed with TBST (Tris-buffered saline [TBS] containing 0.1% Tween 20; Sigma-Aldrich) and blocked for 1 h with 5% BSA–TBS or 5% nonfat dry milk. Subsequently, the membranes were incubated with one of the primary antibodies diluted in 5% BSA–TBS buffer at 4°C overnight. After thorough washing with TBST, the membranes were incubated with secondary antibodies conjugated to a specific fluorochrome (DyLight 800; Thermo Fisher Scientific) or horseradish peroxidase (HRP-conjugated polyclonal anti-mouse/anti-rabbit immunoglobulins; Dako) diluted 1:5,000 in 5% nonfat dry milk–TBST for 1 h at room temperature. The chemiluminescence reaction was developed with the Clarity Western ECL system (Bio-Rad). For GAPDH detection, hFAB rhodamine anti-GAPDH primary antibody (Bio-Rad) was used. Specific protein bands were detected using the ChemiDoc MP imaging system (Bio-Rad).

### Immunostaining.

For staining of intracellular proteins, cells were seeded onto 12-mm round glass coverslips, which were previously washed in 60% ethanol–40% HCl, thoroughly rinsed with water, and sterilized. After stimulation with interferon or viral infection at the desired time points, cells on the coverslips were washed with PBS and immediately fixed with 4% formaldehyde (20 min at room temperature). Cells were then washed three times with PBS and incubated with 100% cold methanol for 20 min at −20°C. After washing with PBS, coverslips with cells were blocked and permeabilized for 1.5 h with 5% BSA (Sigma-Aldrich) with 0.3% Triton X-100 (Sigma-Aldrich) in PBS at room temperature. After the removal of the blocking solution, coverslips with cells were incubated with primary antibodies diluted in PBS containing 1% BSA and 0.3% Triton X-100 overnight at 4°C. After the cells were washed five times with PBS, the appropriate secondary antibodies conjugated with fluorescent dyes were added, and the cells were incubated for 1 h at room temperature. Subsequently, the cells were washed, and their nuclei were stained with 200 ng/mL 4′,6-diamidino-2-phenylindole (DAPI) (Sigma-Aldrich) for 10 min. After a final wash in MilliQ water, coverslips with stained cells were mounted onto microscope slides with a drop of Mowiol (Sigma-Aldrich). The cellular sublocalization of stained proteins was observed using a Leica SP5 confocal microscope and Leica Application Suite AF software.

### Data analysis.

**(i) Image analysis.** Confocal images obtained from immunostaining were analyzed using our in-house software (https://pmbm.ippt.pan.pl/software/shuttletracker). Nuclear outlines were detected based on DAPI staining. Outlines of nuclei that were partially out of frame or mitotic were excluded from the analysis; outlines of overlapping nuclei were split based on geometric convexity defects when possible. Cells were classified as either RSV negative or RSV positive based on the combination of the sum of the intensities of the pixels inside nuclear outlines (weight of 5) and the perinuclear region (weight of 1, decreasing sigmoidally with distance from the nuclear contour). Cells were classified as either IAV negative or IAV positive based on the sum of the intensities of pixels inside nuclear outlines.

**(ii) Credible interval.** Credible intervals (CrIs) of the Kolmogorov-Smirnov statistics (Fig. 3C and D, Fig. 7B, and Fig. 8C and D) were estimated by resampling data points 10^5^ times. For each resampling, data points were drawn at random with probabilities proportional to the number of data points in each category (virus positive and virus negative), and the number of resampled data points was equal to the total number of data points in both categories.
